# Supplementary material for: Structural and magnetic properties of core-shell Au/Fe3O4 nanoparticles
Source: Sci Rep. 2017 Feb 6;7:41732. doi: 10.1038/srep41732 (PMC5292710; doi:10.1038/srep41732)
Supplement: Supplementary Information [file srep41732-s1.pdf]

Structural and magnetic properties of core-shell Au/Fe_3O_4 nanoparticles

Lizbet León F. [1,2], J. A. H. Coaquira [1], M. A. R. Martínez [1], G. F. Goya [2], J. Mantilla [1], M. H. Sousa [3], L. de los Santos Valladares [4], C.H.W. Barnes [4] and P. C. Morais [1,5]

[1] Laboratory of Magnetic Characterization, Instituto de Física, Universidade de Brasília, DF 70910-900, Brasília, Brazil.
[2] Instituto de Nanociencia de Aragón (INA), Universidad de Zaragoza, 50018 Zaragoza, Spain.
[3] Green Nanotechnology Group, Faculdade de Ceilândia, Universidade de Brasília, Ceilândia, DF 72220-900, Brasília, Brazil.
[4] Cavendish Laboratory, Department of Physics, University of Cambridge, J.J Thomson Av., Cambridge CB3 0HE, United Kingdom
[5] School of Chemistry and Chemical Engineering, Anhui University, Hefei 230601, China

Correspondence and requests for materials should be addressed to L. de los Santos Valladares (ld301@cam.ac.uk)

Supplementary material

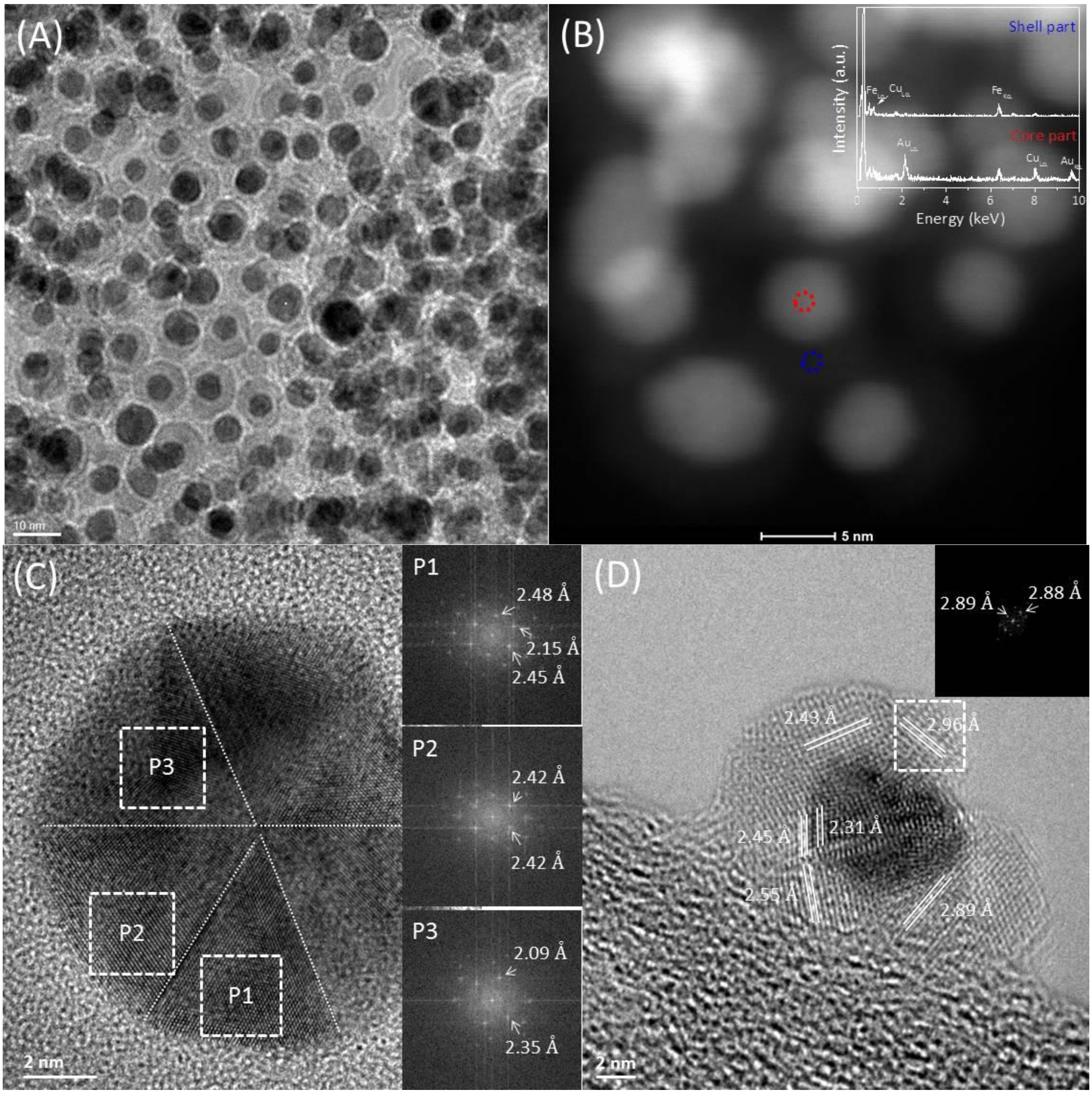

Fig. S1

Transmission electron microscopy (TEM) images of the core-shell Au/Fe_3O_4 nanoparticles. a) TEM images of dispersed particles. b) Dark-field TEM image. The inset shows the EDS spectra acquired from the marked areas that confirms the presence of Au core and shell magnetite (Cu and C peaks are due to carbon copper grid). c) HR-TEM of a Au nanoparticle and electrons diffraction patterns obtained from selected areas and d) HR-TEM of an individual Au-Fe_3O_4 core-shell NP and its respective electron diffraction patters (inset). The faceted surface of the Au core match well to the

observed fringes of 2.89, 2.55, 2.45, 2.43 and 2.96 Å thicknesses which correspond to the (022), (113), (222), (222) and (022) reflections of the spinel structure of the magnetite (the 2.31 Å thickness fringe with (111) reflection corresponds to the gold core). The inset EDS comes from the highlighted region with fringes 2.89 and 2.88 Å and corresponds to the (111) and (022) reflection of the magnetite.

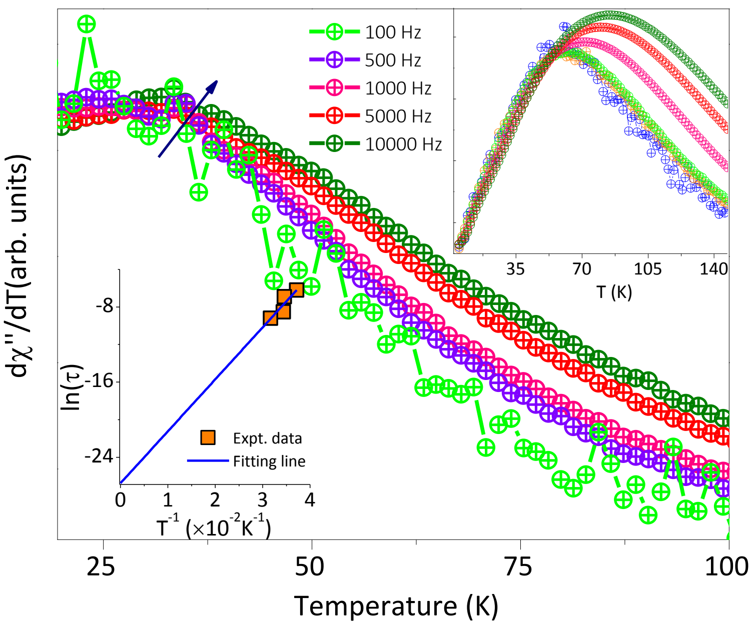

Fig. S2

Temperature dependence for the out-of-phase component of the ac magnetic susceptibility of the ferrofluid Au/Fe_3O_4. Curves were obtained for increasing excitation frequencies at fixed amplitude of 5 Oe. Main panel shows the derivative of the χ"(T) curves from Fig. 4 that present a maximum at lower temperatures. The upper right panel shows the χ’’vsT plot. The lower-left panel shows the plots of the relaxation time as a function of the inverse of T_m. The solid line is the fit with a Néel-Arrhenius relation.
